# “If there is a tension about something, I can solve it”: A qualitative investigation of change processes in a trial of brief problem‐solving interventions for common adolescent mental health problems in India

**DOI:** 10.1111/papt.12433

**Published:** 2022-11-09

**Authors:** Kanika Malik, Rachana Parikh, Rooplata Sahu, Paulomi Sudhir, Christopher G. Fairburn, Vikram Patel, Daniel Michelson

**Affiliations:** ^1^ Sangath New Delhi India; ^2^ Jindal School of Psychology and Counselling O.P. Jindal Global University Sonipat India; ^3^ Department of Clinical, Neuro and Developmental Psychology Vrije Universiteit Amsterdam The Netherlands; ^4^ PATH New Delhi India; ^5^ Department of Clinical Psychology National Institute of Mental Health and Neuro Sciences Bengaluru India; ^6^ Department of Psychiatry University of Oxford Oxford UK; ^7^ Department of Global Health and Social Medicine Harvard Medical School Boston Massachusetts USA; ^8^ Department of Global Health and Population Harvard TH Chan School of Public Health Boston Massachusetts USA; ^9^ School of Psychology University of Sussex Brighton UK; ^10^ Department of Child and Adolescent Psychiatry Institute of Psychiatry, Psychology and Neuroscience King's College London London UK

**Keywords:** adolescents, India, mental health, problem solving, qualitative, schools

## Abstract

**Objectives:**

There is limited understanding of change processes and long‐term effects of low‐intensity psychosocial interventions. We investigated these aspects in two brief problem‐solving intervention formats for adolescents with elevated mental health symptoms and associated distress/impairment.

**Methods:**

This qualitative study was nested within a school‐based randomized controlled trial in New Delhi, India, which compared two problem‐solving intervention formats: a lay counsellor‐led format supported by printed materials (intervention arm) and printed problem‐solving materials alone (“bibliotherapy” control arm). A total of 32 participants, ranging in age from 14 to 20 years (mean = 16.4 years, *SD* = 1.9) and comprising 21 males and 11 females, were interviewed across both trial arms at 12‐month follow‐up.

**Results:**

Five themes were derived using thematic framework analysis. The “impacts on symptoms and functioning” theme described symptomatic improvements and functional gains. “Processes underlying problem solving” reflected changes in positive beliefs, attitudes and emotions when confronted with problems, and the use of a more effective problem‐solving coping style. “Experiences of problem‐solving materials” covered benefits (e.g. access to relatable stories and readymade solutions) and limitations (e.g. diminishing use over time) of printed problem‐solving handouts. “Role of supporting figures” accounted for the facilitating roles played by counsellors and trusted others. There were also accounts of researchers functioning as *de facto* counsellors in the bibliotherapy arm. “Recommended modifications for intervention delivery” included more flexible and private ways to access the interventions, greater personalization of the counselling process, more engaging and relevant supporting materials, and suggestions for widening access to the interventions in schools and community settings.

**Conclusions:**

We infer from our qualitative analysis that changes in problem‐solving style and problem orientation underpinned long‐term symptomatic and functional improvements. Participants in the counsellor‐led intervention appeared better able to sustain the use of problem‐solving skills and generalize this approach beyond the original presenting problems. We attribute the differences between arms to the influence of direct advice and supportive interactions with counsellors. Practice implications are discussed.

**Practitioner points**

- Participants in two problem‐solving intervention formats (bibliotherapy and counsellor‐led problem solving) reported an array of symptomatic and functional improvements, which in many cases were sustained over the 12‐month follow‐up period.
- Symptomatic and functional improvements appeared to be driven by changes in participants' problem orientation (i.e. developing a more positive mindset about addressing problems) and problem‐solving style (i.e. developing knowledge and skills that result in more effective “solutions” to problems).
- Changes in problem‐solving processes were facilitated by supportive interactions and directive guidance from lay counsellors; even brief interactions with researchers were experienced positively by participants in the self‐directed bibliotherapy condition.
- “Ultra‐brief” human support could be a viable and credible option for improving outcomes of self‐directed psychosocial interventions, especially in low‐resource school settings where suitably trained providers – including those from non‐specialist backgrounds – may be in very short supply.
- The study also highlighted implementation supports (e.g. ways to ensure privacy, methods to schedule sessions and reminders, and storage logistics for student‐facing materials) that require attention within school environments.

## INTRODUCTION

A growing body of global evidence attests to the effectiveness of “low‐intensity” psychosocial interventions for common adolescent mental health problems (Catanzano et al., 2020; Orygen, The National Centre of Excellence in Youth Mental Health, 2018). Compared to conventional psychotherapies, low‐intensity interventions are less resource‐intensive due to their typically brief schedules (usually not more than six sessions) and involvement of less highly specialized practitioners (e.g. paraprofessionals or “lay” counsellors) in delivery roles (Shafran et al., 2021). Many of these interventions employ a pared down number of practice elements (i.e. discrete clinical techniques or strategies within a larger intervention plan), which target common mechanisms across diverse problem types. Such “transdiagnostic” protocols have the potential to streamline delivery and enhance utility when applied to real‐world case presentations, which are typically characterized by a high degree of comorbidity (Chorpita et al., 2020; Michelson & Patel, 2017). Transdiagnostic approaches have garnered particular attention in low‐ and middle‐income countries (LMICs), where human resources are especially scarce and broad‐based treatments may be more amenable to scaling up (Murray et al., 2019).

The “PRemIum for aDolEscents” (PRIDE) programme in India has developed a school‐based, transdiagnostic stepped care architecture to address anxiety, depression and conduct problems, which together account for over 75% of the adolescent mental health burden globally (Erskine et al., 2015). The systematic developmental process involved scoping literature reviews, formative qualitative studies with adolescents and other relevant stakeholders, and extensive piloting (Michelson, Malik, Krishna, et al., 2020). Problem solving was selected as a first‐line practice element, reflecting its frequent use in evidence‐based intervention protocols for common adolescent mental health problems (Hogue et al., 2020; Michelson et al., 2022). Problem solving is also a key skill with direct relevance to psychosocial stressors that were prioritized in adolescents' explanatory models of distress in our formative research (Parikh, Michelson, Sapru, et al., 2019). Consistent with stress‐coping theory (Lazarus & Folkman, 1984), the therapeutic focus on problem‐solving skills is intended to rebalance the transactional stress‐coping system in two ways: (i) by modifying “problem orientation” from a negative to a more positive stance, and (ii) by teaching an adaptive “problem‐solving style” where structured steps can be used to define and resolve problems effectively (Michelson et al., 2022).

The problem‐solving component of the PRIDE programme (denoted as “Step 1” within the stepped care architecture) was tested in a randomized controlled trial during 2018–2019. Detailed descriptions of the trial design and main findings have been published elsewhere (Malik et al., 2021; Michelson, Malik, Parikh, et al., 2020; Parikh, Michelson, Malik, et al., 2019). In brief, the trial was implemented with 250 adolescents who presented with elevated mental health symptoms and associated distress/impairment in six government‐run schools serving low‐income communities in New Delhi, India. Participants were randomly allocated to one of two low‐intensity problem‐solving formats: a counsellor‐led version, supported by printed problem‐solving materials (intervention arm), or a “bibliotherapy” version comprised solely of the printed problem‐solving materials (control arm). In the counsellor‐led format, problem solving was introduced in 4–5 sessions using a three‐step heuristic (P‐O‐D, referring to “problem” identification, generating “options,” and “do it” in the real‐world), followed by a review of efforts to put solutions into practice and extend the POD steps to other problems. Participants received three “POD booklets” and a “POD poster” that further explained and reinforced the use of problem‐solving steps through comic strip stories and written homework exercises. Bibliotherapy participants in the control arm received the same POD booklets for self‐study accompanied by brief printed instructions, but without accessing a counsellor. In line with wider evidence suggesting that interventions with human support may be more effective than bibliotherapy or other purely self‐help formats (Bennett et al., 2019), the counsellor‐led format showed small but sustained effects on self‐reported psychosocial problems at short‐term follow‐up (6 and 12 weeks) and over 12 months. The trial additionally demonstrated significant long‐term effects of the counsellor‐led format on mental health symptoms, functional impairment and perceived stress. There were also long‐term changes in the bibliotherapy arm, although these were smaller in magnitude than the changes associated with the counsellor‐led problem‐solving format (Malik et al., 2021; Michelson, Malik, Parikh, et al., 2020).

The breadth and durability of these outcomes are striking, given the brevity of the problem‐solving intervention formats and the use of a single‐element design. Additionally, mediation analysis indicated that a measure of perceived stress (partly related to the construct of problem orientation) at 6 weeks mediated 15% and 23% of the intervention group effects on measures of psychosocial problem severity and mental health symptoms, respectively, at 12 weeks (Michelson, Malik, Parikh, et al., 2020). Looking beyond the PRIDE trial, a recent review identified only five randomized controlled trials that tested standalone problem‐solving interventions for young people with depression or anxiety, none of which included a fully powered mediation analysis (Michelson et al., 2022).

The complementary use of qualitative methods has the potential to further elucidate mechanisms of change, for example, with respect to the potential relational enhancement provided by peer counsellors and through a detailed exploration of problem‐solving style and how it might be influenced by different intervention materials. There are also implications of such process‐oriented research for the wider field of mental health science, where increasing attention has focused on identifying core “active ingredients” in youth psychotherapies, referring to “those aspects of an intervention that drive clinical effect, are conceptually well defined, and link to specific hypothesised mechanisms of action” (Wolpert et al., 2021). In line with this trend, an in‐depth analysis of how participants experience problem solving within a parsimonious, single‐element intervention design may offer valuable insights that can help to build more precise and efficient interventions in future.

The current qualitative study was embedded within the PRIDE Step 1 trial and incorporates interviews that were collected by the trial's research team shortly after the 12‐month outcome assessments. The specific research questions were as follows: (i) What did participants consider to be the main impacts of problem‐solving interventions delivered in low‐intensity counsellor‐led and bibliotherapy formats? (ii) How did specific content and delivery strategies affect outcomes and engagement for participants in the respective intervention formats? (iii) What content and delivery modifications were suggested by participants to optimize effectiveness and scalability of the problem‐solving interventions?

## METHODS

### Design and setting

The original PRIDE trial protocol was modified on the recommendation of the Trial Steering Committee to include a *post‐hoc* extension of the endpoint assessments (originally at 6 and 12 weeks) to 12 months. The current qualitative study formed part of the protocol modification and was approved by the Institutional Review Boards of Harvard Medical School (sponsor) and Sangath (implementing organization in India). Informed assent (with additional parent/guardian consent) was obtained from all participants below the age of 18 years; adolescents aged 18 years and above provided consent directly.

Qualitative interviews were deemed to be an appropriate qualitative data collection method, keeping in mind our interest in exploring participants' subjective experiences of the respective interventions (Clarke & Braun, 2013), and the lack of validated quantitative measures of problem‐solving mechanisms in the study setting (Michelson et al., 2022). To help with structuring the descriptions of qualitative methods and findings, we followed Consolidated Criteria for Reporting Qualitative Research (Tong et al., 2007). A completed COREQ checklist is available in Appendix S1, specifying the locations of key details related to study design, research team, reflexivity, analysis, and findings.

### Sampling and recruitment

The overall sampling frame in the current study included 176 adolescents who were followed up at 12 months as part of the host trial (95 from the intervention arm; 81 from the control arm). We considered the principle of “information power” (i.e. the more information power the sample provides, the smaller the sample size needs to be; Malterud et al., 2016) when arriving at a target sample size of approximately 30 participants for the current study. This estimate took into account the relatively specific aims of the qualitative sub‐study, the spread of sample characteristics, the expected quality of interviews (based on our experience of conducting other qualitative research with adolescents in the study context [Gonsalves et al., 2021; Michelson, Malik, Krishna, et al., 2020]), our cross‐case analysis plan, and application of established theory (Lazarus & Folkman, 1984; Michelson et al., 2022). We used a mixed sampling strategy that initially involved consecutive recruitment of adolescents who opted‐in for a qualitative interview after completing the 12‐month outcome assessment in the host trial. After recruiting half of the intended qualitative sample, purposive sampling was deployed to increase diversity in terms of age, gender and intervention engagement.

The final qualitative sample consisted of 32 participants. This included 21 adolescents who received the counsellor‐led format in the original host trial, most of whom (*n* = 18, 86%) were classed as remitted by 12 months; and 11 adolescents who received bibliotherapy, out of which six (55%) were remitted. Participants were aged from 14 to 20 years (mean age = 16.4 years, *SD* = 1.9), with the majority being males (*n* = 21, 65%).

### Data collection

A semi‐structured interview guide (Appendix S2) was developed to explore (i) subjective perceptions of improvement for the main presenting problems and generalized effects on other domains, (ii) processes linking specific intervention content and delivery strategies with perceived outcomes, and (iii) intervention features that could be refined for future implementation. Face‐to‐face interviews were carried out within 1 month after the trial's 12‐month outcome assessment. Interviews were audio‐recorded and conducted at participants' homes and other convenient locations. The mean interview duration was 24.7 mins (*SD* = 7.1).

Interviews were conducted in Hindi by three graduate researchers (two females and one male) who were not involved in intervention delivery during the host trial. The researchers received 5 days of office‐based training in qualitative interviewing. Monthly group supervision involved reviewing audio‐recorded interviews to discuss strengths and areas for improvement (e.g. asking open rather than closed questions, striking the right balance between questioning and listening). All interviews were audio‐recorded and were later transcribed for the purpose of analysis.

### Data analysis

Thematic framework analysis followed five recursive steps involving familiarization, identifying a coding framework, indexing, charting, and mapping and interpretation (Gale et al., 2013; Ritchie et al., 2013). Hindi language transcripts, prepared by professional translators, were read independently by two researchers (KM and RP, both fluent in Hindi and English) to gain an overview of the collected data (familiarization). The bilingual researchers consulted with each other and an English‐speaking senior author (DM) to produce a preliminary coding framework, comprising data‐driven inductive codes and deductive codes that drew on concepts from previous research on problem‐solving processes (Michelson et al., 2022). The coding framework was then applied independently by KM and RP to a subset of transcripts, followed by comparison of codes, and iterative revision of the framework and higher order themes and sub‐themes (indexing). Coded interview fragments were organized into a matrix by theme and sub‐theme. The matrix (prepared using Microsoft Excel) was reviewed regularly with the senior author, leading to further refinements (charting). The final stage (mapping and interpretation) involved operationalizing the thematic labels, comparing across participant groups, developing a narrative interpretation, and selecting illustrative quotes. Quotes were translated from Hindi to English for the purpose of reporting. A copy of the detailed coding framework is provided in Appendix S3.

Our epistemological position recognized that intervention processes are not directly observable but may be inferred through theoretical knowledge and empirical investigation of participants' lived experiences. This is in keeping with a wider tradition of critical realism in which the perception of “ontologically true” reality takes place through the subjective lens of human interpretation (Fletcher, 2016). We did not try to demonstrate inter‐rater reliability but used independent coding to integrate alternate perspectives into the final framework. The bilingual coders had direct experience of the study setting and included a clinical psychologist (KM) who was involved in developing the problem‐solving intervention manual and materials, and a mental health researcher who served as trial coordinator (RP). The wider research team also included senior clinical academics who contributed to the intervention development process. These perspectives facilitated the interpretation of participants' accounts as related to *a priori* theoretical and empirical foundations of the problem‐solving interventions.

## FINDINGS

**FIGURE 1:**
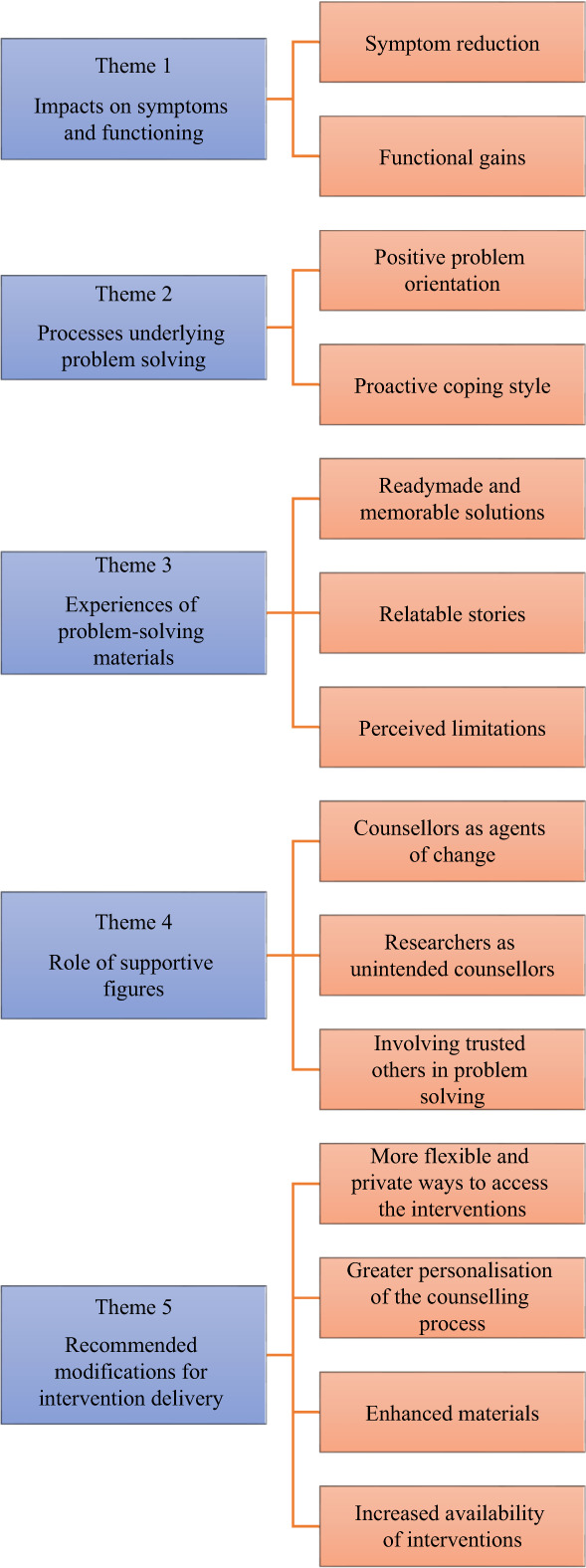
Thematic matrix developed using framework analysis

Figure 1 illustrates the five overarching themes and associated sub‐themes. These are elaborated further below.

### Theme 1: Impacts on symptoms and functioning

Positive impacts were noted by participants across multiple domains, which we grouped into sub‐themes related to changes in symptoms and functioning. Exceptions were also noted where participants described non‐remitted and recurring difficulties.

#### Symptom reduction

Participants in both arms identified reductions for a variety of emotional and behavioural symptoms, including “lowered levels of fear,” “decreased worries,” “control over anxiety while communicating with others,” “better mood,” “decreased anger outburst,” “lesser fights” and “better temper control.” In most cases, participants related these impacts to focal problems that were identified at the outset of the problem‐solving intervention.

I am not as irritable anymore when I am with my friends or family, and we speak cordially now. We don't quarrel as often. (P21, male, grade 9, counsellor‐led format)

I am confident now. And [I have learned that] I shouldn't isolate myself. Before this, when I would feel tense, I would stay alone, and that would increase the tension. But now, I don't isolate myself. (P31, female, grade 12, bibliotherapy)

There were also accounts of partial remission, where participants acknowledged the benefits of using a problem‐solving approach but felt that improvement could be further strengthened with time and practice.

Earlier, I would go completely blank if I was interacting with a group of people. I could not decide whether I should talk, what should I talk about, what shoul say, but now I am not like that… I talk, I try and socialise with them, I laugh and talk. It has become easier, but I am still a little hesitant; it's not gone. But I think it will get better with the passage of time. (P12, female, grade 12, counsellor‐led format)

#### Functional gains

Many participants described improvements in their social relationships, reflected in descriptions of “getting on much better,” “communicating effectively,” and “able to enjoy time” with friends and family.

Counselling has made a lot of difference. I couldn't have thought that problems can be solved like this… Not only are my problems resolved, but also my relationships with others have improved. (P22, male, grade 10, bibliotherapy)

My issues were impacting my family, and I fought with family members all the time. For anything that they would say, I would feel bad, get angry and fight with them. Now, that does not happen. They have learned to do things slowly with me, and I also take their comments casually. (P16, male, grade 12, counsellor‐led format)

One participant described how the relationship with their father had improved after the latter made direct use of problem‐solving materials to address their own anger difficulties.

One day, I dropped a glass of water, and my father started scolding me. Despite my mother's efforts to cool him down, he got angrier. Later, I said, ‘Papa, don't be angry. I got a booklet from my school, and it is written in the booklet, that if a person gets angry, what they can do.’ My father read [the booklet], then cooled down. Now my father doesn't unnecessarily scold me. (P11, female, grade 9, counsellor‐led format)

In terms of academic functioning, participants noted improvements in focus, efficiency and productivity, which in some cases appeared to be secondary to the resolution of cognitive symptoms and motivational deficits.

I can now study well. My papers went well too a my mind remains fresh. My mind is set on studying well for my exams. (P11, female, grade 9, counsellor‐led format)

I can now concentrate on my studies, since I have reduced the use of phone and fighting with others… This has [postively] impacted my school performance, and I am scoring better marks now. (P22, male, grade 10, bibliotherapy)

Two participants who used the bibliotherapy format suggested that their problems had resolved because of external factors beyond their control (e.g. school curriculum changes; relocating with family to a new home). Apart from these exceptions, all other study participants attributed functional gains to the effects of the intervention. However, four participants (two in each format) described persistent problems that were unchanged over time.

There is no improvement yet, and there continues to be the problem with studies. I can only remember for some time, and then I forget [what I studied]. (P23, male, grade 12, bibliotherapy)

When I was taking the counselling, I had many options to improve my studies, based on the tips shared by sir [counsellor]. And I was able to study – be it with my sisters or with other kids who would come to my home for tuition. I would study with them and also teach them. Then [post‐counselling] I left it all and got addicted to smartphone and social media like WhatsApp, Facebook, and YouTube. (P15, male, grade 11, counsellor‐led format)

### Theme 2: Processes underlying problem solving

This theme reflected participants' accounts of how a problem‐solving approach helped them to mitigate the negative impacts of internal and external stressors. In line with stress‐coping theory (Lazarus & Folkman, 1984), participants described changes in both problem orientation and problem‐solving style. Changes in problem orientation indicated a more constructive and hopeful mindset, where problems can be seen as manageable challenges rather than pervasive or uncontrollable threats. Changes in problem‐solving style were marked by a shift towards proactive coping for well‐defined target problems, as opposed to an avoidant style with diffuse problem targets. These facets of problem solving are summarized as distinct though related sub‐categories below.

#### Positive problem orientation

Participants from both arms of the trial indicated a shift from a negative orientation to a more positive stance in which they felt more confident about managing problems now and in the future.

After undergoing this [counselling], I think I would not experience such difficulties again in future, but if I experience any major difficulties, I can work on it myself using the POD steps. (P14, male, grade 12, counsellor‐led format)

If there are any problems, even if they may appear serious, we need to be able to solve them, especially if we are stuck alone with the problem… we can use these books and try to solve our problems. (P26, male, grade 9, bibliotherapy)

#### Proactive coping style

Participants in both arms described how the problem‐solving approach encouraged them to use more proactive coping strategies rather than letting problems build up.

My problems were focusing on academics and managing time [for studying]; both are much better now. It was most helpful when the [counsellor] said, ‘You should make a timetable.’ I made a timetable, and I benefited a lot from it. I followed it and [even after a year] still do it whenever I feel there is high [work] load. It is going very well. (P1, male, grade 9, counsellor‐led format)

I am beginning to understand that in this situation, I can control myself, discuss and arrive at a solution instead of getting angry. And if I can discuss it normally, the situation may resolve faster. (P23, male, grade 12, bibliotherapy)

While participants from both arms discussed the use and benefits of problem solving when applied to their original presenting problems, examples of generalizing problem‐solving skills proactively to other problems over the 12‐month period were restricted to the counsellor‐led format.

If there is a tension about something, I can solve it with this [POD steps]. This has helped me prepare notes when studying, improve relationships with friends, and build on my communication skills. Many situations can be worked out through these [steps], from family matters to everything else. (P21, male, grade 9, counsellor‐led format)

[During counselling] I would make a plan and then work on it. So, I would develop 6–7 options – so if one didn't work, I could work on another. So, similarly, in future, when needed, I will make a plan and develop options and implement them one‐by‐one [to solve a problem]. (P11, female, grade 9, counsellor‐led format)

### Theme 3: Experiences of problem‐solving materials

This theme encompassed participants' views about the benefits and limitations of printed materials as an aide to problem solving.

#### Readymade and memorable solutions

Participants in both arms indicated that they appreciated how the booklets served as a “solution bank” that offered specific solutions to common problems, as well as offering structured descriptions of problem‐solving steps. The P‐O‐D heuristic (“Problem – Options – Do it”) was perceived as being particularly memorable and helpful.

In the booklets, it was explained how to relax oneself, I followed that and learned ways to calm and relax myself. (P32, male, grade 10, bibliotherapy)

I found the title ‘POD’ as most useful in working through problems… The booklets said that first, see what our problem is and understand it. Then, develop the ways in which we can solve the problem. Then ‘do it’ – meaning try them and see. (P14, male, grade 12, counsellor‐led format)

And the booklets helped a lot because they made me realise that I need to bring some change in my thoughts… If there are problems, then we should understand them, and only after understanding our problems can we find a solution. (P24, female, grade 10, bibliotherapy)

#### Relatable stories

Participants also commented favourably on the motivating effects of reading relatable stories. The comic style formatting with easy‐to‐read text in vernacular language further helped adolescents to engage with these booklets.

There was a story in the book about Priyanka. When I read the story, what she did, then how she came out, I saw all that… I did hesitate a little even after discussion with madam [counsellor], but then I read the stories, then I saw what happened in it, then I did the same and felt better. (P4, female, grade 9, counsellor‐led format)

Booklets had a lot of information, which was quite helpful for me. I realised I have similar problems as the story's characters. It also helped me understand that I can improve upon them. (P23, male, grade 11, bibliotherapy)

#### Perceived limitations

Participants in both arms described how they made more extensive use of the materials at the outset, but relatively few participants continued to use the booklets after the first month. Several participants reported that they had lost the POD booklets and the poster by the time of the 12‐month research interview.

Yes sir, I read it on the same day when I had received it and then again after 3‐4 days. After that, I didn't have the time to read it, and I had also misplaced the books. (P23, male, grade 12, bibliotherapy)

Other frequently cited reasons for not using printed materials in the longer term included not experiencing any new problems, materials not being helpful for the current problems, lack of time due to academic demands, and not liking the writing exercises in the booklets. Some of the participants in the counsellor‐led format reported that they did not feel the need to revisit booklets since they remembered the steps from counselling.

### Theme 4: Role of supportive figures

Participants frequently described empathic and trusting interactions with counsellors (in the counsellor‐led format) and research staff (in the bibliotherapy format) that lent credibility to the problem‐solving materials, and in the case of the counsellor‐led format, provided personalized advice about how to apply problem‐solving to specific problems. Some participants also described supportive roles of trusted family members in applying problem solving to the home environment.

#### Counsellors as agents of change

Participants in the counsellor‐led format described how interactions during face‐to‐face sessions encouraged them to open up about their problems.

He [counsellor] helped me as a friend, spoke nicely with me and encouraged me to share all my problems openly. He [counsellor] said, ‘I would help you solve them [problems], so I told him everything without fear.’ So, after that, he [counsellor] told me all the good ways to solve the problem, and then I kept working on them. (P9, male, grade 10, counsellor‐led format)

Many participants spoke approvingly of receiving directive advice from counsellors, reflecting adolescents' preferences for concrete “tips” that could be applied to their problems.

[My counsellor] would tell me things. I would think about it and try them. I was feeling better, as things were improving. Like, for my academic‐related problems, ma'am [my counsellor] had asked me to make a timetable for every minute of the day – for going out with friends, eating, drinking, and studying. I did that, and I am more attentive in my studies today. (P1, male, grade 9, counsellor‐led format)

Then I spoke to sir [my counsellor] and he spoke to me in a very good manner. After that, I followed what he told me. This made it easier for me… Whatever he taught me, I followed it as it is, and that has helped me a lot! (P9, male, grade 10, counsellor‐led format)

Participants also described how counsellors helped them navigate through the POD booklets and clarify written concepts.

When I read the booklet, I could not understand what I had to do. Then I asked [my counsellor], ‘Ma'am, what do I have to do in this, I do not understand.’ Ma'am explained everything to me, what I had to do and how. Then I completed it as instructed. (P21, male, grade 9, counsellor‐led format)

#### Researchers as unintended counsellors

Several bibliotherapy participants perceived their brief interactions with research staff as a form of counselling. They frequently referred to researchers as “counsellors” or “teachers” and considered their time‐limited interactions to include validating questions and helpful encouragement to use the problem‐solving materials.

Counselling was good, it was good to talk, being asked appropriate questions, and problems being understood [by researcher]. (P23, male, grade 12, bibliotherapy)

The most important difference counselling made [to my life] is that I met a guiding teacher [researcher]. This teacher said that we should read this book, it will help us, and we should follow this [book]… If I hadn't come to counselling, I wouldn't be able to think like that. (P22, male, grade 10, bibliotherapy)

#### Involving trusted others in problem solving

Some participants in the counsellor‐led format also mentioned the importance of involving trusted others (e.g. parents and siblings) in the problem‐solving process. The participants deemed this additional support to be useful for identifying solutions and overcoming barriers to implementing chosen strategies.

I found it difficult to generate the options. Like, if I have generated a particular solution, I would ask my sister to tell me if that solution was right for my situation. I was not able to do it alone. (P14, male, grade 12, counsellor‐led format)

However, a larger number of participants preferred to keep their experiences of problem‐solving private, as they were apprehensive of negative reactions and lack of support from others.

Madam, what is the use of discussing this with someone else, for they will not understand it correctly and later make fun of it. So, I did not take anyone's help. (P1, male, grade 9, counsellor‐led format)

### Theme 5: Recommended modifications for intervention delivery

Modifications recommended by the participants were grouped into four sub‐themes, as set out below.

#### More flexible and private ways to access the interventions

Some participants in the counsellor‐led format explained that a lack of flexibility around session timings caused them to miss classes, which irritated their teachers. Additional concerns were raised about methods for calling students out of class to attend intervention sessions, which often attracted undesirable attention from peers and curiosity about the participants' problems. Accordingly, participants recommended that students should be given more agency when scheduling counselling sessions as well as “drop‐in” options. Other privacy‐related recommendations focused on providing discreet locations for counselling sessions and offering storage for printed problem‐solving materials at school. The latter was viewed as a way to prevent unwanted visibility at home. Meetings outside of school were generally discounted, again for reasons of privacy.

There should be a secluded space [for sessions], where no one else should be able to hear us talk, and it should be just the two of us. Some students bring their friends while waiting [for sessions]; this should not be allowed either. (P11, female, grade 9, counsellor‐led format)

They [friends] would say that – ‘you are going for counselling, you are disturbed in your mind, or there should be a problem. So, definitely there is some problem which is why you are going for counselling.’ It was like torture. (P23, male, grade 12, bibliotherapy)

#### Greater personalization of the counselling process

Participants in the counsellor‐led format highlighted the importance of the counsellor being responsive to their particular needs and preferences. For some participants, this was discussed in relation to the need for a directive approach when preparing to use problem‐solving steps outside of sessions.

Like to me, [when] the counsellor said, ‘make a timetable,’ I did not make it. You have to guide them, make them read, make them practice with you, then [the student] will definitely do it. (P5, female, grade 9, counsellor‐led format)

Several participants, including some who received bibliotherapy, discussed the importance of explaining concepts in clear and personally relatable terms.

I can read the booklets myself, but it would be good if the person sitting opposite to me [the researcher] also explained them. They can explain them in their own way, what are these things, and we can go about solving our problem. (P32, male, grade 10, bibliotherapy)

#### Enhanced materials

Participants suggested various modifications to the printed materials, such as using more “mature” looking character graphics, combining three booklets into a single volume, and offering additional reflective exercises in the booklets. It was also suggested that engagement could be improved through the use of humour and introducing videos in place of, or in addition to, booklets.

In terms of content, participants expressed clear preferences for a greater number of readymade “solutions” that could be applied to current and future problems. Several participants felt that interpersonal issues related to friends and family were relatively under‐represented in the illustrated stories. There were corresponding recommendations for more relevant and realistic stories involving interpersonal scenarios and advice about handling interpersonal challenges.

There should have been two or three more stories on different problems, so if any other problem comes, then we should be able to use booklets. (P4, female, grade 9, counsellor‐led format)

#### Increased availability of interventions

Participants recommended that problem‐solving interventions could be made routinely available throughout the academic year and offered as a universal “life skills” intervention.

[Counsellors] are not available in school through the year, [but] they should be available… I think all students should get some counselling. There should be a dedicated period of counselling like we have for other subjects, which will help more students reach out for help. They [counsellor] have offered counselling to existing students, but since they are not there, many new students don't get the chance. (P17, male, grade 9, counsellor‐led format)

We should have a counsellor permanently in the school, like a teacher… So, if there is a permanent counsellor, then all the [students] can share their problems with them readily. It's not like there are just 1 or 2 students [who need help], there are thousands. (P23, male, grade 12, bibliotherapy)

Some participants in both arms proposed that the interventions should be extended to younger age groups and implemented on a wider scale across schools.

This counselling programme took place in a few schools of Delhi. I think this should be implemented in all schools. Expanding these services will help adolescents like me, who otherwise get very little support from others in working through problems. I think the counselling will benefit all. (P1, male, grade 9, counsellor‐led format)

## DISCUSSION

We investigated therapeutic impacts and change processes for adolescent participants approximately 12 months after enrolling in a randomized controlled trial of two low‐intensity problem‐solving formats in New Delhi, India. Qualitative interviews with a sub‐sample of trial participants suggested that both counsellor‐led and bibliotherapy formats helped adolescents to identify and apply proactive solutions for specific stressors, which contributed to a hopeful mindset and adaptive style for managing present and future challenges. Consistent with stress‐coping theory (Lazarus & Folkman, 1984), we deduce that changes in problem‐solving style and problem orientation appeared to underpin long‐term symptomatic relief and functional improvements, especially in the counsellor‐led format where direct advice about potential solutions and warm and empathic interactions with counsellors encouraged engagement with problem‐solving tasks. This potentially facilitated participants' ability to generalize problem‐solving skills beyond their initial presenting problems.

The current findings complement quantitative evidence from the host trial, which demonstrated significant and sustained advantages of the counsellor‐led intervention over 12 months (Malik et al., 2021; Michelson, Malik, Parikh, et al., 2020). The wider literature on low‐intensity interventions has also shown that human facilitation is associated with improved mental health outcomes and engagement over purely self‐directed formats (Bennett et al., 2019). We found that directive guidance was especially valued in our sample, acting as a key facilitator for problem solving. This is consistent with previous studies from the PRIDE research group and others across the world, showing that young people favour helping professionals who can provide relatable, age‐appropriate information to help with understanding their problems along with concrete advice about coping strategies (Cooper et al., 2021; Gonsalves et al., 2019; Mcarthur et al., 2016; Michelson, Malik, Krishna, et al., 2020; Persson et al., 2017). Preferences for more active therapist input have been interpreted in other research as a sign of readiness to change and as a marker of problem “assimilation,” where problems have been brought into conscious awareness and can be actively resolved (Stiles, 2002). These preferences for directivity can also be understood in the context of cultural expectations around didactic learning, which have been described in educational and psychotherapeutic contexts in India (Agrahari, 2016; Manickam, 2010).

The caring and empathic skills of counsellors were also highly valued by the adolescent participants. While these relational processes are not specific to problem‐solving interventions, previous research shows that information shared during a psychosocial intervention is more likely to be taken on board and act as a spur for behaviour change when delivered in the context of an effective working alliance (Freake et al., 2007; Radez et al., 2021). Interviews from the bibliotherapy arm suggested that even brief interactions with researchers were valued and had a motivating effect on participants who received an ostensibly self‐guided problem‐solving intervention. A similar “non‐specific” effect of research assessments has been observed in other studies, where participants have described the benefits of warm and empathic interactions with researchers, irrespective of the specific intervention(s) that were being trialled (Fowler et al., 2021; Sanchez et al., 2022). This highlights the need to account for the unintended effects of research assessments, for example, by utilizing self‐reported outcome measures that can be administered independently of research staff. Our findings also raise the possibility that ultra‐brief human facilitation could offer incremental benefits over self‐help interventions that are completely self‐guided. Future work on low‐intensity interventions should examine other potentially resource‐efficient ways of providing human guidance, for example, through shared decision‐making protocols to decide on optimal dosing and blending human guidance with digital support (Gonsalves et al., 2021; Malik et al., 2021).

Parents and other family members played important roles in facilitating change for some participants, by acknowledging the credibility of the printed materials, reviewing the steps of problem solving, and providing specific suggestions about potential solutions. On the other hand, many participants preferred not to share problem‐solving materials or even reveal their involvement to family or peers for reasons of stigma. This serves as a reminder that adolescents are not a singular group and mental health interventions must acknowledge individuals' preferences for privacy and autonomy (Cardy et al., 2020; Radez et al., 2021). As shown in previous research, stigma‐related concerns can be mitigated, in part, by direct assurances about confidentiality and the development of trust in relationships with intervention providers (Gronholm et al., 2018). On a practical level, we noted specific recommendations from participants in the current study that counselling sessions should be offered in discreet locations in schools, and with flexible timings that allow students to attend outside of regular classes. Some participants were also reluctant to take printed materials home and recommended that storage space should be provided on school premises.

Consistent with previous qualitative research and lived‐experience reports (Michelson et al., 2022), findings from the current study suggest that the procedural clarity of problem solving and its practical focus on here‐and‐now solutions make this element especially suited to a first‐line youth mental health intervention. Comparing across participants, it was not obvious that any demographic group was more or less likely to benefit from a focus on problem‐solving skills. Rather, there was a general preference for personalization (e.g. tailored advice about how to approach individual problems). The use of a stepped protocol where personalized practice elements follow on from problem solving is being addressed in other studies from the PRIDE programme and was not specifically considered in the present study (Chorpita et al., 2020; Malik et al., 2022).

Overall, experiential accounts and participant recommendations in this study indicated that problem solving is a credible approach that could conceivably be offered at scale in low‐resource school settings in India. Future scale‐up should consider involving adolescents, parents and school leaders in identifying implementation features that are practical and acceptable for delivery within the logistical constraints of the school environment (Gee et al., 2021).

### Study strengths and limitations

The current investigation is among the few studies that have explored processes of change and longer term impacts of a low‐intensity psychosocial intervention aimed at young people. When considered alongside the host trial results (which lacked a fully powered mediation analysis; Malik et al., 2021; Michelson, Malik, Parikh, et al., 2020), the qualitative evidence fraom participants' first‐hand accounts helps to advance understanding of how and why intervention participants experienced positive and negative outcomes. Furthermore, by sampling from both trial arms, we have been able to compare across conditions and explore differences and similarities in change processes, whereas previous process evaluations have tended to prioritize one intervention arm only (Amos et al., 2019; Haller et al., 2019).

We also acknowledge several study limitations. We did not systematically collect data on those trial participants who were approached but declined to take part in qualitative interviews and it is possible that our sample may have been skewed towards more highly engaged participants. However, we note that over three quarters of the counsellor‐led format participants were completers in both the host trial and the current nested qualitative study.

Our team lacked “outsider” perspectives that might have offered alternative insights during data analysis and interpretation. In particular, our interest in developing effective problem‐solving interventions may have inadvertently led to an interpretive bias that minimized negative attributes of the problem‐solving approach and emphasized positive attributes. That said, we deliberately recruited participants with varying levels of intervention engagement and our topic guide specifically probed negative intervention experiences. Nevertheless, we acknowledge that the credibility of the findings could have been strengthened further through the use of participatory approaches to analysis, such as respondent validation (Sellars et al., 2021). Future intervention research should engage directly with the lived experience of participants to co‐construct knowledge that represents adolescents' voices as fully and accurately as possible.

Our study was designed to explore processes of change at the individual level and within the adolescent–counsellor dyad for two low‐intensity problem‐solving intervention formats. There is also a need for research, informed by implementation science principles, that engages more fully with the wider context of intervention delivery (e.g. factors affecting fidelity to the intervention model). Such evidence is vital for developing implementation strategies that can bolster the external validity of the interventions, while ensuring fidelity to the main theorised active ingredient of problem solving (Bauer & Kirchner, 2020).

## CONCLUSIONS

The findings from this study add to the evidence base on the impacts of low‐intensity psychosocial interventions for common adolescent mental health problems and underlying change processes. Participants in two low‐intensity problem‐solving intervention formats described a range of symptomatic and functional improvements driven by changes in underlying problem orientation and problem‐solving skills. Participants in the counsellor‐led format valued a directive approach to learning and applying problem solving, which appeared to help with generalizing problem‐solving skills beyond the original focal problems. Although there was a general preference among adolescents that favoured more human support, the findings also showed that even a one‐off interaction with a trusted adult can have motivating effects and enhance the credibility of self‐directed materials. Future work is needed to understand how to optimize the amount of human support available in first‐line interventions, and thus inform decision‐making about when and how to offer more intensive support (e.g. for participants with refractory problems).

## AUTHOR CONTRIBUTIONS

**Kanika Malik:** Study design, data management, data analysis, staff supervision, study administration, manuscript drafting. **Rachana Parikh:** Data analysis, manuscript drafting. **Rooplata Sahu:** Data management, staff supervision, study administration, manuscript review. **Paulomi Sudhir:** Study conception, manuscript review. **Christopher G. Fairburn:** Study conception, manuscript review. **Vikram Patel:** Funding acquisition, study conception, study design, manuscript review. **Daniel Michelson:** Study conception, study design, data analysis, staff supervision, critical review and editing of manuscript.

## FUNDING INFORMATION

This research was funded by a Wellcome Trust Principal Research Fellowship grant to VP (106919/Z/15/Z), https://wellcome.ac.uk. The funder had no role in study design, data collection and analysis, decision to publish or preparation of the manuscript.

## CONFLICT OF INTEREST

Authors declare no conflict of interest.

## Supporting information


Appendix S1.

Appendix S2.

Appendix S3.

## Data Availability

The detailed coding framework used in this study is available on request from the corresponding author or PRIDE programme PI. Participants did not provide permission to share raw data (i.e. unprocessed transcripts) outside of the core research team.
