# Supplementary material for: “If there is a tension about something, I can solve it”: A qualitative investigation of change processes in a trial of brief problem‐solving interventions for common adolescent mental health problems in India
Source: Psychol Psychother. 2022 Nov 9;96(1):189–208. doi: 10.1111/papt.12433 (PMC10099760; doi:10.1111/papt.12433)
Supplement: Supplementary file 2 — Appendix S2. [file PAPT-96-189-s002.pdf]

Interview schedule for long-term follow up

Hi, I am *[state your name and role in the project]*. I am working with my team members to understand the long-term impact of school counseling on students lives. Last year you participated in the PRIDE program that took place in your school and through today's interview I would like to understand the impact of counselling in your life over the last year. We would like to know your honest feedback, as this will help us to improve our program. If it is okay with you, I will be audio-recording our conversation. The purpose is to get all the details of what you are saying without having to take too many notes. That way, I can give you my full attention. I assure you that the recording will be kept confidential and will not be shared with anyone else without your permission – unless there is information that suggests your safety is at risk. If that were to happen, then we will discuss it with you.

The entire procedure can take up to 25-30 minutes. If you have any questions, please ask me now or stop me at any point.

Last year you sought help for your problems [state problems from YTP], I would like to know how you are doing with respect to these.

1. What changes have you observed in the problems that you reported to the counsellor last year?
    - What problems have improved?
    - What problems have not improved or even gotten worse?
    - How much are the original problems affecting important areas of your life at the moment (e.g. family, friendships, school, hobbies)?
2. What difference, if any, has counselling made to these problems and how they affect your life?
3. [INTERVENTION ARM ONLY] What activities or lessons do you recall from the meetings with the counsellor? Of the various activities/lessons you described, what are the one or two things that have been most helpful to you?
    - How often – and in what situations – have you used these activities/lessons since counselling finished?
    - When was the last time that you thought about or used activities/lessons from counselling?
    - How much/where do you expect to use these activities/lessons in the future?
4. [INTERVENTION ARM ONLY] What activities/lessons from counselling have been less helpful to you? Can you think of anything that might have made these parts of counselling more helpful?
5. What do you recall about the booklets that were given to you?
    - What, if anything, was helpful about the booklets?
    - Do you still have them? If yes, where do you keep them? When was the last time you used them?
    - What difficulties, if any, did you face while using the booklets? Can you share some examples of situations where you faced difficulties in using them?
6. What other kinds of help you have sought for your original problems? How helpful they have been in learning to manage your problem?

7. What suggestions do you have about how to improve the school counselling program?
   - [INTERVENTION ARM ONLY] If you could change anything about the meetings with the counsellor, what would it be and why?
   - How can the booklets be made better?
   - What kind of additional help/ inputs might improve the experience and results of counselling?
8. Can you think of any negative or unwanted effects of counselling?
   - Has anyone said or done anything negative towards you as a result of your involvement in counselling?
   - How comfortable did you feel/would you feel to discuss negative/unwanted effects with another person? What difference did/would this make?
9. If further counselling was available now, would you like to seek help again?
   - If yes, what kind of help/ inputs will you seek in counselling? The same as before? Different and how?
   - If no, can you describe the reason for not wanting further counselling?

WRAP UP

Thank you for taking the time to meet with me today. We value your time and opinion. Before we wrap up, is there anything else that you would like to tell me that I haven't already asked about? Do you have any additional questions or concerns
