# Supplementary material for: “If there is a tension about something, I can solve it”: A qualitative investigation of change processes in a trial of brief problem‐solving interventions for common adolescent mental health problems in India
Source: Psychol Psychother. 2022 Nov 9;96(1):189–208. doi: 10.1111/papt.12433 (PMC10099760; doi:10.1111/papt.12433)
Supplement: Supplementary file 3 — Appendix S3. [file PAPT-96-189-s001.docx]

**Appendix 3**

**Coding framework used for analysis**

| **Themes with definition** | **Sub-themes** | **Summarised codes** | **Summarised sub-codes** |
| --- | --- | --- | --- |
| Impacts on symptoms and functioning:  *In line with intervention’s focus, this theme encompassed changes in focal emotional and behavioural symptoms and extended impact on functionality in areas such as relationships and academic performance.* | Symptoms reduction | Improvements in emotional symptoms | Positive/ partial/ lack of thereof |
|  |  | Improvement in behavioural symptoms | Positive/ partial/ lack of thereof |
|  | Functional gains | Improvements in social relationships | Positive/ partial/ lack of thereof |
|  |  | Improvements in academic functioning | Positive/ partial/ lack of thereof |
| Processes underlying problem solving:  *In line with stress-coping theory^[[1]](#footnote-2)^, this theme covered change processes in participant’s problem orientation and coping style.* | Positive problem orientation | More positive stance towards solving problems | Present/absent |
|  |  | Confident about managing problems | Present/absent |
|  | Proactive coping style | Applying to presenting problems | Present/absent |
|  |  | Generalizing to other problems | Present/absent |
| Experiences of problem-solving materials:  *This theme encompassed the benefits and limitations of printed materials as an aide to problem solving.* | Readymade and memorable solutions | Benefits of “solution bank” | Present/absent |
|  |  | Benefits of POD acronym | Present/absent |
|  | Relatable stories | Engaging material | Present/absent |
|  |  | Motivating effect | Present/absent |
|  | Perceived limitations | Diminishing use over time | Not experiencing any new problems/ materials not being helpful/ lack of time/ disliked writing exercises |
|  |  | Did not feel the need to revisit booklets | Present/absent |
| Role of supportive figures:  *This theme comprised role of counsellor and other supportive figures in facilitating solving of problems among adolescents.* | Counsellors as agents of change | Warm and empathic interactions | Facilitative/no impact |
|  |  | Directive advice from counsellors | Facilitative/no impact |
|  |  | Helped to navigate through POD booklet | Facilitative/no impact |
|  | Researchers as unintended counsellors | Researchers perceived as “counsellors” or “teachers” | Present/absent |
|  | Involving trusted others in problem solving | Involved trusted others | Facilitative/no impact |
|  |  | Did not involve others | Apprehension about privacy/ not being supported |
| Recommended modifications for intervention delivery:  *This theme comprised programme components that could be optimised to have a greater impact among adolescents in schools.* | More flexible and private ways to access the interventions | Flexibility around session timings | Discreet methods for calling students/ more agency to students in scheduling sessions |
|  |  | Discreet locations for counselling | Session/ storing material |
|  | Greater personalisation of the counselling process | Responsiveness to participants’ needs and preferences | Present/absent |
|  |  | More guidance time, if needed | Present/absent |
|  | Enhanced materials | Improvement in content | More readymade solutions/ use of humour/ use of videos/ more reflective exercises |
|  |  | Improvement in design | Mature looking character graphics/ three booklets into a single volume |
|  | Increased availability of interventions | Available throughout the academic year | Present/absent |
|  |  | Extended to younger age groups | Present/absent |
|  |  | Implemented on a wider scale across schools | Present/absent |

1. Lazarus, R. S., & Folkman, S. (1984). Stress, Appraisal, and Coping. Springer Publishing Company. [↑](#footnote-ref-2)
